# Rapid-onset obesity, hypoventilation, hypothalamic dysfunction, autonomic dysregulation and neuroendocrine tumor syndrome with a homogenous enlargement of the pituitary gland: a case report

**DOI:** 10.1186/s13256-016-1116-z

**Published:** 2016-11-22

**Authors:** Lama Aljabban, Lina Kassab, Nour Alhuda Bakoura, Mohammad Fayez Alsalka, Ismaeil Maksoud

**Affiliations:** 1 Genetic Diseases Unit, Pediatric Department, Faculty of Medicine, Damascus University, Damascus, Syrian Arab Republic; 2 Pediatric Department, Faculty of Medicine, Damascus University, Damascus, Syrian Arab Republic; 3 Faculty of Medicine, Damascus University, Damascus, Syrian Arab Republic; 4 Genetic Diseases Unit, Damascus University Children Hospital, Almouasa Square, Almazzeh, Damascus, Syrian Arab Republic

**Keywords:** Case report, ROHHAD, Obesity, Hypothalamic dysfunction, Ganglioneuroma, Pituitary gland

## Abstract

**Background:**

Rapid-onset obesity with hypoventilation, hypothalamic dysfunction, and autonomic dysregulation syndrome is a rare pediatric disorder with a variable sequence of clinical presentations, undefined etiology, and high risk of mortality. Our patient presented an unusual course of the disease accompanied by a homogenous mild enlargement of her pituitary gland with an intact pituitary–endocrine axis which, to the best of our knowledge, represents a new finding in rapid-onset obesity with hypoventilation, hypothalamic dysfunction, and autonomic dysregulation syndrome.

**Case presentation:**

We present a documented case of a 4 years and 8-month-old Syrian Arabic girl with a distinctive course of signs and symptoms of rapid-onset obesity with hypoventilation, hypothalamic dysfunction, and autonomic dysregulation syndrome accompanied by mature ganglioneuroma in her chest, a homogenous mild enlargement of her pituitary gland, generalized cortical brain atrophy, and seizures. Three months after her first marked symptoms were noted she had a sudden progression of severe respiratory distress that ended with her death.

**Conclusions:**

The findings of this case could increase our understanding of the pathogenetic mechanisms of rapid-onset obesity with hypoventilation, hypothalamic dysfunction, and autonomic dysregulation, and place more emphases on pediatricians to consider rapid-onset obesity with hypoventilation, hypothalamic dysfunction, and autonomic dysregulation syndrome whenever early rapid onset of obesity, associated with any malfunction, is observed in children. This knowledge could be lifesaving for children with rapid-onset obesity with hypoventilation, hypothalamic dysfunction, and autonomic dysregulation syndrome.

## Background

Rapid-onset obesity with hypoventilation, hypothalamic dysfunction, and autonomic dysregulation (ROHHAD) is a rare pediatric disorder. It was first described in 1965 as late-onset central hypoventilation with hypothalamic dysfunction (LO-CHH) [1].

Rapid hyperphagic gain of weight in a previously normal child after 2 years of age is the most consistent first symptom of ROHHAD [2]. Respiratory signs and symptoms may include obstructive sleep apnea, snoring, cyanosis, and alveolar hypoventilation causing hypoxemia and hypercarbia [3], which may cause cardiorespiratory arrest and even sudden death before being recognized [4].

Evidence of hypothalamic dysfunction is defined by the presence of one or more of the following findings: rapid onset obesity, hyperprolactinemia, central hypothyroidism, central diabetes insipidus, hypernatremia/hyponatremia, failed growth hormone stimulation test, corticotropin deficiency, or delayed or precocious puberty [5]. Autonomic dysregulation may manifest as altered pupil response to light, strabismus, gastrointestinal dysmotility (constipation or diarrhea), thermal dysregulation (hyperthermia/hypothermia), decreased sensation of pain, tachyarrhythmia or bradyarrhythmia, altered sweating, or hypertension [6, 7]. Other features in one subset of patients may include behavioral disorders, developmental disorders, intellectual impairment, and seizures which need to be closely evaluated to be sure that episodes are not related to hypoxemia or hypernatremia [8–10]. Approximately 35% of patients with ROHHAD have neural crest tumors (ganglioneuroblastomas and ganglioneuromas) in the chest or abdomen at a median of 2.4 years interval following the onset of hypothalamic dysfunction [11]. The modified acronym ROHHAD-NET (ROHHAD-neuroendocrine tumor) is used when a neural crest tumor is present [3].

## Case presentation

Our case is a 4 years and 8-month-old Syrian Arabic girl with a birth weight of 4 kg, who is the sixth of seven siblings born to healthy consanguineous parents. She followed normal psychomotor development and experienced no remarkable illness until the age of 4 years and 2 months when her parents noticed a rapid weight gain (about 1 kg every 10 to 15 days) due to excessive eating (she required six to seven big meals/day). Later, they observed her to have alterations in body secretion (decreased tears when crying, decreased nasal discharge, unfavorable body odor, and decreased sweating) in addition to blue cold extremities, diarrhea alternated with constipation, polyuria, and polydipsia. Two months after the onset of obesity she had urinary incontinence during night sleep. Then, significant behavioral changes developed including: mood alteration, anxiety episodes, rage attacks, nervousness, and aggressive behavior, in addition to recurrent fatigue, social withdrawal, prolonged periods of sleep (12 hours continuously), and difficulty staying awake during the day. These complaints grew to be a serious concern to the family so she was admitted to Damascus Children Hospital, endocrinal department, at the age of 4.5 years, for further investigations.

For three generations, the family history was negative for similar presentations, obesity, or psychiatric disorders. It was noteworthy that an older sister of our patient had died at the age of 12 years with a diagnosis of acute myeloid leukemia (AML). Her sister’s malignancy was not accompanied by any of the signs or symptoms our patient had. 

**Fig. 1:**
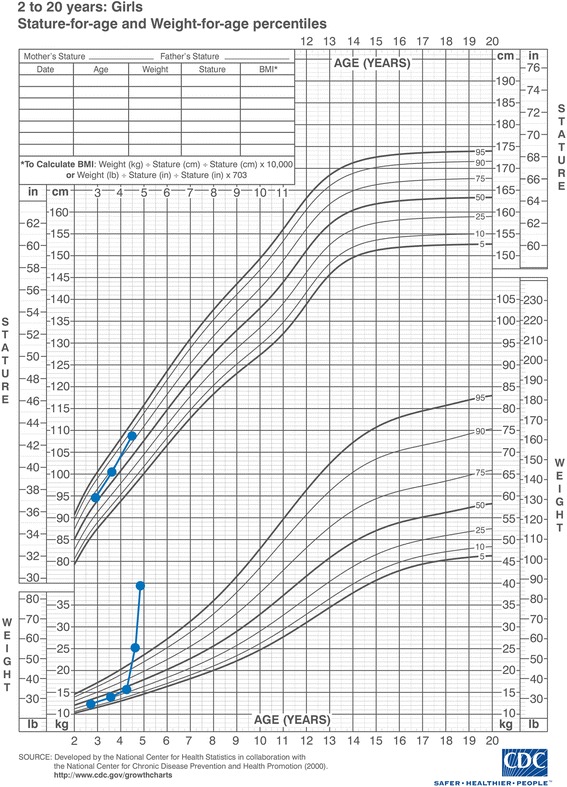
A growth chart showing stature and weight for age

**Fig. 2:**
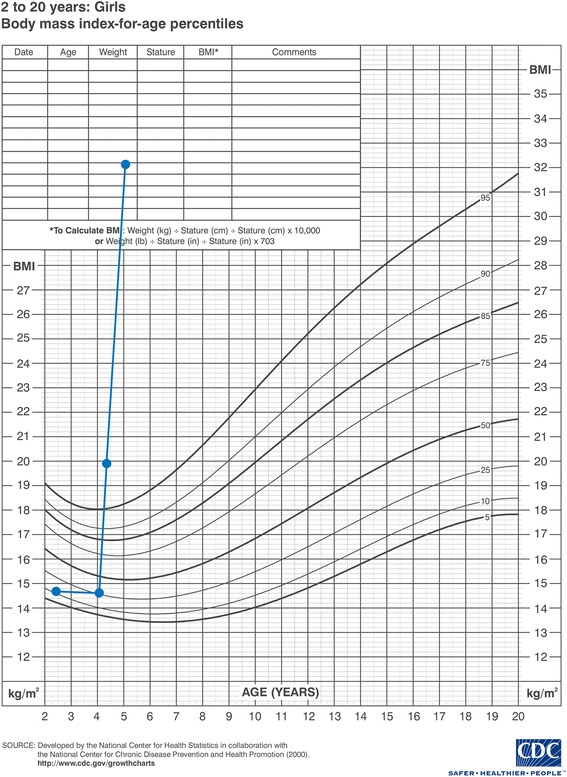
A growth chart showing body mass index for age

On physical examination, general obesity was noticed without striae or altered skin pigmentation. No dysmorphic features were observed, neither were there any minor or major congenital malformations. Her weight was 25 kg (above 97% percentile) while her length and head circumference measured 110 cm (at 90% percentile) and 52 cm respectively. Her body mass index (BMI) was equal to 20.1 (Figs. 1 and 2).

An ophthalmic examination revealed left exotropia with normal fundus and normal papillary response. The performed laboratory analyses did not indicate any significant abnormality. Her complete blood count (CBC), blood glucose, sodium (Na), chlorine (Cl), potassium (K), calcium (Ca), alanine aminotransferase (ALT), aspartate aminotransferase (AST), triglycerides (TG), cholesterol, urine density, creatinine, and urea were all within normal limits. Her thyroid-stimulating hormone (TSH), free thyroxine (T_4_), morning cortisol, adrenocorticotropic hormone (ACTH), prolactin, growth hormone (GH), insulin-like growth factor-1 (IGF1), follicle-stimulating hormone (FSH), and luteinizing hormone (LH) were also within normal limits.

**Fig. 3:**
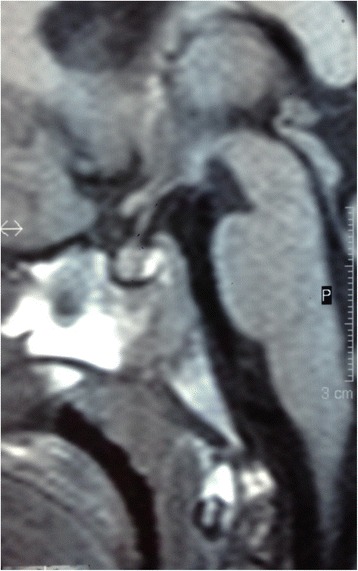
Magnetic resonance imaging. Sagittal and coronal precontrast T1-weighted image of the patient’s pituitary gland showing normal anterior and posterior lobes, with homogeneous enlargement and convex upper surface of the gland

**Fig. 4:**
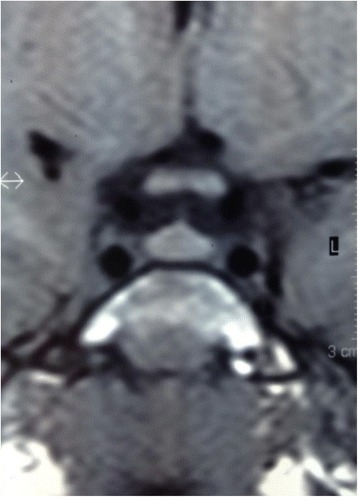
Magnetic resonance imaging. Sagittal and coronal precontrast T1-weighted image of the patient’s pituitary gland showing normal anterior and posterior lobes, with homogeneous enlargement and convex upper surface of the gland

Her abdominal and pelvic ultrasound was normal. Her bone age was slightly advanced and it fitted 5 to 5.5 years old. Brain magnetic resonance imaging (MRI) was normal; it showed no cortical atrophy. A homogenous mild enlargement of her pituitary gland was observed on both T1-weighted and T2-weighted images with normal hyperintense posterior lobe on T1-weighted images (Figs. 3 and 4).

**Fig. 5:**
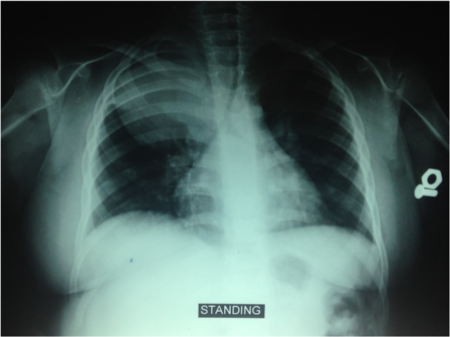
Chest X-ray reveals a big round opacity in the right lung

Since the endocrine function tests were normal, her symptoms had been explained on the basis of psychotropic origin, and she was discharged with antipsychotic medication (risperidone 1 mg orally per day) and the recommendation to follow up at the out-patient psychiatric clinic after 3 months of prescribed treatment. After 3 months of discharge, when she was 4 years and 8 months old, she developed dyspnea that worsened with exertion and during sleep, continuous snoring, and recurrent chest pain. The respiratory symptoms lasted for 5 days after which she had two episodes of cyanosis and obstructive apnea which lasted for 10 minutes with improvement via oxygen mask. As a result of progressive respiratory distress (respiratory rate 35/min, expiratory grunting, flaring of nostrils, wheezing with prolonged expiration, and remarkably loud snoring) she was readmitted to Damascus Children Hospital, intensive care unit (ICU). Investigations showed hypoxemia, hypercapnia, and respiratory acidosis accompanied by radiological evidence of a big round opacity in her right lung (Fig. 5).

She was intubated 24 hours after admission and attached to a mechanical ventilator because of severe respiratory distress with altered consciousness: blood gases were pH 7.27, partial pressure of carbon dioxide (PaCO_2_) 79 mmHg, partial pressure of oxygen (PaO_2_) 38 mmHg, and oxygen saturation 79%. Her laboratory results were within normal limits except for elevated values of Na (up to 162) and C-reactive protein (77 mg/L). During this admission, her weight was recorded as 40 kg (>97th percentile).

**Fig. 6:**
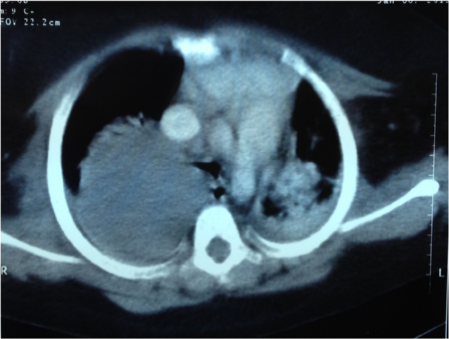
Chest computed tomography scan showing a mass in the right lung

A chest computed tomography (CT) scan demonstrated a 6 cm round mass filling most of her right lung and pushing her heart and mediastinum to the left with infiltration and consolidation of the right lower lobe of her right lung (Fig. 6).

**Fig. 7:**
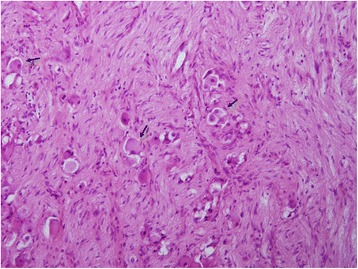
Microscopic view of the mass tissue stained by hematoxylin and eosin showing mature elements: ganglion cells (*black arrows*) and neurites accompanied by Schwann cells and fibrous tissue (20×)

Subsequently, when her health stabilized, a complete resection was performed of a 10×10 cm solid round mass from the posterior wall of her chest. It was a mature ganglioneuroma (Fig. 7).

Her unexplained rapid gain of weight and the presence of chest ganglioneuroma brought attention to ROHAAD syndrome as a diagnosis of her situation and helped to exclude other illnesses that could be considered in the differential diagnosis. Familial obesity and Prader–Willi syndrome were both excluded since her family history did not support the first, and with the absence of mental retardation and congenital abnormalities there was no indication to investigate the latter.

**Fig. 8:**
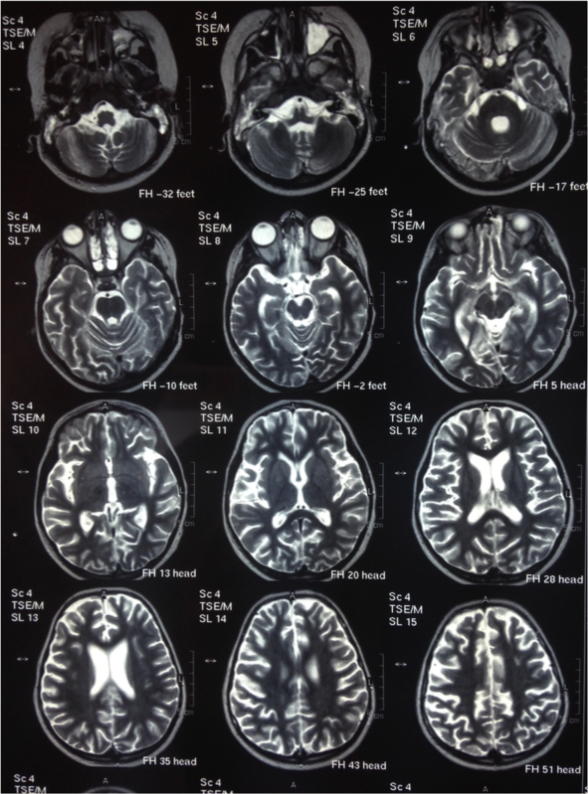
Brain magnetic resonance imaging sections showing generalized brain atrophy

A good improvement in consciousness was achieved by stopping sedative drugs, so she was able to speak, move her limbs, and interact with others. Nevertheless, she required prolonged ventilation because of respiratory instability and she had myoclonic seizures. Three attempts at extubation failed without a clear reason. A brain MRI was ordered which revealed a generalized cortical atrophy of her brain with the same mild pituitary gland enlargement observed in a previous MRI (Fig. 8).

**Fig. 9:**
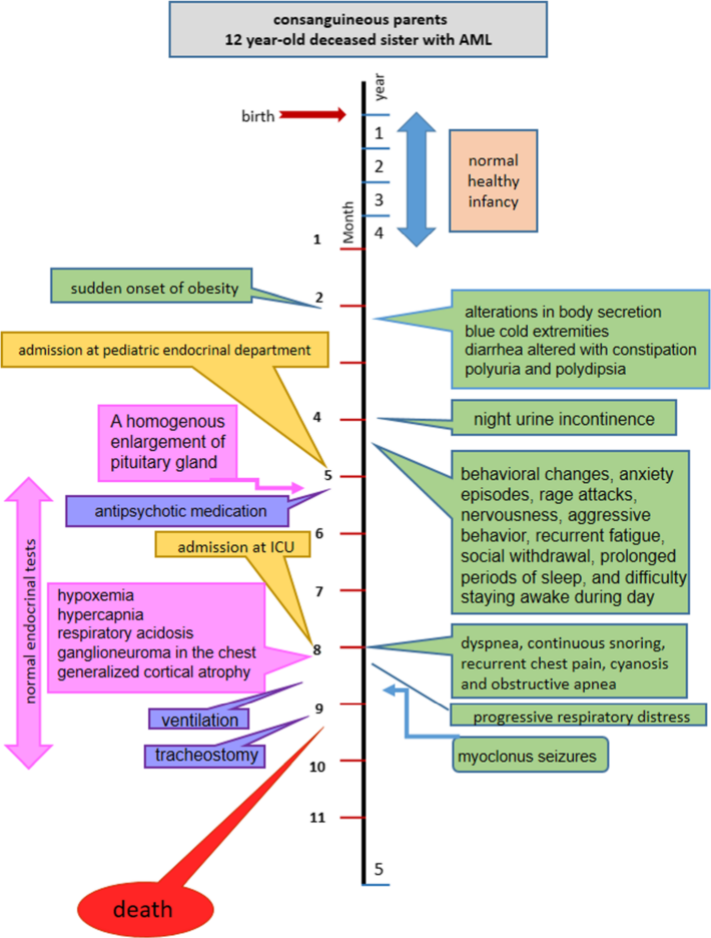
Timeline of signs and symptoms

A month after her admission to the ICU, a tracheostomy was performed because of lack of spontaneous breathing. One week later, she had cardiorespiratory arrest and died. A timeline of her signs and symptoms is shown in Fig. 9.

## Discussion

ROHHAD is a very rare disease with less than 100 reported cases worldwide to-date [12]. The course and timing of ROHHAD symptoms are largely variable. Our patient showed many features of ROHHAD. The symptoms appeared as a new course of timing and presentation. She had a normal healthy childhood for 4 years and 2 months after which she developed a rapid onset of obesity. At the age of 4.5 years, many symptoms related to an autonomic dysregulation and a psychiatric disturbance developed without accompanying respiratory or endocrinal findings.

After only 3 months she developed alveolar hypoventilation that progressed to severe respiratory distress and led to mechanical ventilation at our ICU. During the course of her signs and symptoms her pituitary–endocrine axis seemed to be intact and we observed four neurological findings: a mild enlarged pituitary gland, a neural crest tumor, generalized brain atrophy, and myoclonic seizures.

Her pituitary gland measured 7 mm in height and 11 mm in width, with convex upper surface (Fig. 3). These findings fitted the gland of a teenaged female [13, 14]. Usually, the height of the pituitary gland measures less than 6 mm in children under the age of 12 years with a flat or slightly concave upper surface [15, 16]. Premature puberty was ruled out since no clinical signs nor hormonal findings pointed to it. There is a strong suggestion that ROHHAD syndrome could share similar pathogenetic mechanisms with other immune-mediated central nervous system disorders, but many aspects remain to be explained regarding the etiopathogenesis. A paraneoplastic etiology has been suggested mainly because of the frequent association with neural crest tumors, but recent studies failed to confirm this role [17]. Tumors of neural crest origin are well known to trigger paraneoplastic phenomena, including conditions such as opsoclonus-myoclonus syndrome [10]. The neural findings in our patient could support the paraneoplastic etiology of ROHHAD with the emphases that she did not record any episode of hypoxia while under monitoring in our ICU and that the removal of the tumor did not improve her situation. It was also postulated that an autoimmune phenomenon precipitated by specific environment exposures could underline the etiology of ROHHAD [18].

The fact that our patient has a first-degree relative with AML and consanguineous parents brings to mind the hypothesis of ROHHAD being an epigenetic condition [10, 19].

LO-CHH used to be confused with ROHHAD because of the overlapping presentations. Nevertheless, patients with ROHHAD syndrome express a wide spectrum of hypothalamic and autonomic abnormalities unlike patients with LO-CHH [5]. In addition, genetic evaluation of the LO-CHH-causing mutations in the Paired-like homeobox 2b (*PHOX2B*) gene that encodes a transcription factor in the autonomic nervous system, established the distinction between these two clinical disorders [19]. Molecular studies showed that sequencing of the *PHOX2B* gene was normal in children with ROHHAD [8]. Further studies of candidate genes suspected to be involved in the etiology of ROHHAD based on their role in the embryologic development of the hypothalamus and autonomic nerve system [10], or in neuronal development [18], failed to define any genetic determinant of ROHHAD [8]. At the present time, no diagnostic biomarker is available to confirm the diagnosis of ROHHAD which is absolutely based on clinical criteria. At this stage, the decisive clinical recognition of ROHHAD could be lifesaving and could prevent the patient from dramatic consequences due to the severity of clinical manifestations. ROHHAD syndrome is considered a life-threatening medical condition with death occurring around the average chronological age of 10 [11]. Of children with ROHHAD, 25% are reported to die because of respiratory failure [6]. Of patients with ROHHAD, 50% have obstructive cyanotic episodes and obstructive sleep apnea followed by symptoms of abnormal respiratory control before cardiorespiratory arrest [2].

## Conclusions

It was suggested that ROHHAD syndrome should be considered in all cases of rapid and early-onset obesity associated with hypothalamic pituitary endocrine dysfunctions [7]. Since the course of clinical presentations is very variable and unpredictable, and because endocrinal symptoms may not be found in some patients like ours, or may develop late after years of manifested alveolar hypoventilation [9], we suggest that any rapid gain of weight in an otherwise healthy child after the age of 2 should be regarded and treated as ROHHAD syndrome.

## Abbreviations

ACTH: Adrenocorticotropic hormone
ALT: Alanine aminotransferase
AML: Acute myeloid leukemia
AST: Aspartate aminotransferase
BMI: Body mass index
Ca: Calcium
CBC: Complete blood count
Cl: Chlorine
CT: Computed tomography
FSH: Follicle-stimulating hormone
GH: Growth hormone
ICU: Intensive care unit
IGF1: Insulin-like growth factor-1
K: Potassium
LH: Luteinizing hormone
LO-CHH: Late-onset central hypoventilation with hypothalamic dysfunction
MRI: Magnetic resonance imaging
Na: Sodium
PaCO_2_: Partial pressure of carbon dioxide
PaO_2_: Partial pressure of oxygen
*PHOX2B*: Paired-like homeobox 2b
ROHHAD: Rapid-onset obesity with hypoventilation, hypothalamic dysfunction, and autonomic dysregulation
ROHHAD-NET: Rapid-onset obesity, hypoventilation, hypothalamic dysfunction, autonomic dysregulation, and neuroendocrine tumor
T_4_: Thyroxine
TG: Triglycerides
TSH: Thyroid-stimulating hormone
